# The Newest Hypothesis about Vitiligo: Most of the Suggested Pathogeneses of Vitiligo Can Be Attributed to Lack of One Factor, Zinc-**α**2-Glycoprotein

**DOI:** 10.5402/2012/405268

**Published:** 2012-06-19

**Authors:** Nooshin Bagherani

**Affiliations:** Nooshin Bagherani's Office, 2nd Floor, Taha Physicians' Building, 40-Meter Street, Khoramshahr, Khuzestan Province, Iran

## Abstract

Zinc-**α**2-glycoprotein (ZAG) is a recently identified adipokine, assigned to the chromosome 7q22.1. It is a multidisciplinary protein, which is secreted in various body fluids. The ZAG plays roles in lipolysis, regulation of metabolism, cell proliferation and differentiation, regulation of melanin synthesis, cell adhesion, immunoregulation, and so forth. Vitiligo is the most common depigmenting skin disorder, characterized by acquired, progressive, and circumscribed amelanosis of the skin and hair. It commonly begins in childhood or young adulthood. The pathogenesis of this disorder is uncertain, but it appears to be dependent on the interaction of genetic, immunological, and neurological factors. For the first time, we pointed the probable association between ZAG and vitiligo. Herein, I have described this association in different views. By confirming this association, a surprising progression will occur in the treatment of this prevalent debilitating disease.

## 1. Introduction of Zinc-***α***2-Glycoprotein

 Zinc-*α*2-glycoprotein (ZAG), a recently identified adipokine [1–4], is a 41,000 Da glycoprotein, secreted by a variety of normal epithelia [5]. For the first time, this 41 kDa single-chain polypeptide [6] was reported in human serum and subsequently purified in 1961 [7]. It was named for its electrophoretic mobility and for its ability to be precipitated by zinc salts [8–10].

 The gene of ZAG, assigned to the chromosome 7q22.1, comprises four exons and three introns [7]. Its first full polypeptide sequence was identified by Araki et al. [11]. The molecule of ZAG has three domains, named *α*1, *α*2, and *α*3 [7]. The amino acids of *α*1 and *α*2 domains are highly conserved [12], indicative of the functional importance of the groove in the biological functions. On the other hand, the *α*3 domain is less conserved, indicative of species specificity and evolutionary constrains [7]. The main function of ZAG gene is the decomposition of fat and reduced fat content [13]. The serum ZAG can easily be detected by tetramethylethylenediamine-enhanced photoluminescent imaging [14].

 There is high degree of similarity between ZAG and antigen-presenting molecules such as class I MHC molecules both at sequence and structural levels. The ZAG is considered as a member of immunoglobulin gene superfamily [7] based on its structure so that its 2.8 angstrom crystal structure [15] has a large groove analogous to class I MHC peptide-binding grooves [7, 12, 15, 16]. Unlike other MHC molecules, ZAG is not membrane bound; so, it appears that its function is different from the peptide presentation and T-cell interaction functions of class I MHC [7, 17]. Moreover, instead of a peptide, the ZAG contains a nonpeptidic compound in the groove, which plays a role in lipid catabolism under normal and pathological conditions [15]. This structure of groove also reflects its role in immunoregulation [7]. Furthermore, it appears that ZAG is the sole soluble member of immunoglobulin gene superfamily that is implicated in cachexia [18].

 The ZAG is a multidisciplinary protein, which is secreted in various body fluids [7] and is found to be ubiquitously and constitutively expressed [18]. Its presence and expression have normally been described in the following sites: normal human fluids such as serum, seminal fluid, saliva, sweat, milk, cerebrospinal fluid, urine and amniotic fluid, kidney extract [7], liver, breast, lung, and prostate [17]. The ZAG is synthesized by adipocytes, liver, epithelial cells of prostate, normal epidermal and buccal epithelia [7]. ZAG secretion by differentiated human adipocytes is abundant [2], and it appears that ZAG has an autocrine/paracrine role in adipose tissue function [17]. In one study on mice, Tzanavari et al. detected ZAG mRNA from one day of birth in subcutaneous fat, which is the main site of ZAG synthesis postnatally. They showed that ZAG mRNA and protein levels fall significantly at weaning and thereafter. At the end, they concluded that the downregulated ZAG expression in white fat depots at weaning, when fat mass is expanded substantially, suggests that ZAG might be involved in the postnatal development of adipose tissue mass [3].

 It appears that renal filtration is an important route for eliminating ZAG; hence, markers of renal function should be considered in studies on ZAG physiology [19].

## 2. Regulation of ZAG Expression

 The gene expression of ZAG is predominately regulated by glucocorticoids [7, 20], androgens, and progestins [7]. The ZAG itself has been revealed to induce ZAG expression in adipocytes [20]. This ability of ZAG in inducing its own expression can explain the much greater effect of ZAG on lipolysis in vivo than its effect on lipolysis in vitro [20, 21].

 Glucocorticoids are able to enhance ZAG expression [20, 22, 23]. The findings suggest that induction of ZAG expression may play role in the action of glucocorticoids. It appears that glucocorticoids induce *β*3-adrenoreceptor in adipocytes by which it amplifies the induction of ZAG expression [20]. Furthermore, eicosapentaenoic acid can downregulate ZAG expression through interference with glucocorticoid signaling [23]. The mechanism by which induction of *β*3-adrenoreceptor leads to an increase in ZAG expression is not known [20, 21].

 It has been shown that the expression of ZAG is increased by the selective *β*3-adrenoreceptor agonist BRL37344, suggesting that the sympathetic system can play a role in the regulation of ZAG expression [20] and that the induction of ZAG is mediated through *β*3-adrenoreceptor [16, 20].

 Interferon-*γ* (IFN-*γ*) is a potential mediator of the increased ZAG expression in cachexia. It has been shown that IFN-*γ* can strongly upregulate expression of ZAG in human epithelial cell lines [20, 24].

 Studies showed that tumor necrosis factor-*α* (TNF-*α*), a major product of macrophages [25], can modulate ZAG expression and secretion by human adipocytes [17, 25]. In one study, Sanders and Tisdale have demonstrated that ZAG expression can be regulated particularly through TNF-*α* and the PPAR-gamma nuclear receptor [26]. TNF-*α* can decrease ZAG expression and secretion [4, 25], while the PPAR-gamma agonist rosiglitazone induces an increase in ZAG level [26]. It appears that the effect of TNF-*α* on ZAG is time-dependent; in other words, this effect appears chronic rather than acute [17]. Studies have shown that proinflammatory cytokines and chemokines such as interleukin-6 (IL-6), leptin, IL-8, MCP-1, and RANTES can upregulate ZAG expression in adipocytes [25].

## 3. Biologic Roles of ZAG in Human

### 3.1. Lipolysis and Lipid Mobilization

 The ZAG is a natural adipocyte factor found in both white and brown adipose tissue [20]. Although the exact role of ZAG in adipose tissue remains to be clarified, it appears that its expression is inversely related to adiposity [27]. In mice it has been revealed that the action of ZAG is associated with downregulated lipogenic enzymes and upregulated lipolytic enzyme expression in adipose tissue [28].

 The ZAG is closely related to obesity [2, 27–29]. Serum ZAG level is inversely correlated with percentage of body fat, fat mass [2, 28, 29], body weight [28, 29], body mass index [2, 28], waist circumference [28, 30], and hip circumference [28]. The ZAG can cause highly significant, time-dependent decrease in body weight. This body weight loss can entirely be attributed to the loss of body fat [21]. Genetic studies have suggested that ZAG may be a candidate gene for body weight regulation [27]. On the other hand, the clearance of ZAG in the circulation may be altered in obesity [17].

 The ZAG is known to promote lipolysis through stimulation of adenylate cyclase in a GTP-dependent process via binding through *β*3-adrenoreceptor [7, 21]. Russell and Tisdale showed that ZAG not only induces direct lipolysis, but also sensitizes adipose tissue to other lipolytic stimuli [31]. Moreover, ZAG via increasing uncoupling protein-1 (UCP-1) expression in brown adipose tissue can lead to lipolysis [21, 26].

 Glucocorticoid-mediated upregulation of ZAG expression in white and brown adipose tissues results in lipolysis [7, 23]; hence, induction of lipolysis by corticosteroids can be attenuated by anti-ZAG antisera [7].

The ZAG is responsible for cachexia or lipid catabolism seen in cancer patients [7, 32]. It appears that this process is due to the upregulation of ZAG secondary to increased level of circulatory glucocorticoids [7, 33].

### 3.2. Regulation of Metabolism

 The ZAG can stimulate energy utilization in adipose tissue and skeletal muscle by upregulation of UCP isoforms and GLUT4. Findings have shown that ZAG can lead to phosphorylation of AMP kinase-*α* and ACC, thereby activating a pathway central to the regulation of energy metabolism [34]. Russell and Tisdale have shown that rats treated with ZAG show a progressive decrease in body weight, without any decrease in food intake, but with a rise in body temperature [31, 35]. They showed that loss of adipose tissue accompany an increase in lean body mass [35]. In another study, they showed that ZAG can decrease phosphorylation of both double-stranded RNA-dependent protein kinase in muscles of diabetic mice; thereby it can attenuate the process of skeletal muscle atrophy. This effect is interestingly shown in gastrocnemius but not soleus muscle [36].

 The circulating ZAG is negatively correlated with fasting insulin, homeostasis model assessment of insulin resistance [2, 19], C reactive protein [2], and leptin [2, 19, 30]. On the other hand, ZAG is positively correlated with adiponectin [2, 30].

 The ZAG plays a role in lipid metabolism [37]. It also can influence adipocyte metabolism locally [2]. Serum level of ZAG is associated with the serum levels of cholesterol [37]. The ZAG genotype appears to be associated with total cholesterol and low-density-lipoprotein cholesterol. ZAG also correlates with glucose, creatinine, and uric acid. Stejskal et al. introduced ZAG as a marker for glucose metabolism [38].

 In detecting of doping with recombinant erythropoietins by isoelectric focusing and western double blotting, ZAG interacts nonspecifically [39].

### 3.3. Fertilization

 The presence of ZAG in human seminal fluid is 6 times more than that in the human serum, which suggests its role in fertilization [7]. The lipid metabolism pathway is a significant mechanism for modulating sperm motility; so, it is predicted that ZAG can play a vital role in sperm motility via such process [7, 40]. On the other hand, ZAG can bind to the sperm membrane and initiate motility which is directly associated to cAMP levels in semen [7]. Ding et al. have demonstrated that ZAG and *α*1-antitrypsin, related to the forward motility of spermatozoa in human seminal plasma, played important roles during maturation of spermatozoa, from the epididymis through fertilization in the female reproductive tract [41].

### 3.4. Regulation of Melanin Synthesis

 The role of ZAG in production of melanin is different. In vitro, increased ZAG can decrease melanin production by decreasing levels of tyrosinase protein and its activity, which are the key step for melanin synthesis. Furthermore, ZAG decreases melanin production in vivo, suggesting that ZAG has a different mechanism in vivo and in vitro. Collectively, it appears that the minimal level of ZAG is required for melanin production [7].

 It has also been shown that ZAG is produced by normal epidermal keratinocytes, where its expression improves with cellular differentiation [7, 42]. It appears that ZAG as a keratinocyte-derived factor influences melanocyte behavior, including melanocyte proliferation, dendricity, and melanin synthesis [7, 43, 44].

 Tumor necrosis factor-*α* (TNF-*α*) also inhibits melanin synthesis by human primary melanoma, but it appears that ZAG inhibits melanin production via a TNF-independent mechanism [7].

### 3.5. Immunoregulation

 Due to high degree of structural similarities to MHC-I, ZAG is considered as a member of immunoglobulin superfamily [7]. The members of this family present peptides to cytotoxic T cells [7, 45]. It has a role in the expression of the immune response [7]. In contrast to MHC-I, ZAG does not require peptides and *β*2-microglobulin for folding [7, 46]. The most plausible role of ZAG in immunity is through its capacity for binding and presenting lipidic ligands to T cells [7]. Although the role of ZAG in immunoregulation cannot be denied, the immune system does not seem to be significantly altered in the absence of ZAG [7, 18].

### 3.6. Cell Adhesion

 ZAG is effective in attaining the properties of cell adhesion between cells and extracellular matrices [7]. This function appears to be due to the presence of Arg-Gly-Asp in *α*3 domain [7, 47]. Integrins require divalent cations such as Mg^2+^ and Mn^2+^ for mediating cell adhesion, and surprisingly, the presence of Mg^2+^ and Mn^2+^ is also needed for cell adhesion by ZAG. This finding describes the role of ZAG in the integrin-mediated adhesion. Other mechanisms for ZAG-mediated cell adhesion have been described [7].

### 3.7. RNase Activity

 It has been shown that the RNase activity of ZAG has similarities to other RNases such as human pancreatic RNase [7, 48]. The RNase activity may be important for the physiologic function of ZAG. In various organs, the biological activity of ZAG is associated with infection, immunoregulation, and antitumor activity. It appears that ZAG as a soluble molecule can degrade and eliminate byproduct of RNA processing reactions within cells, as well as incorrectly synthesized or damaged RNA [7]. Degraded RNA can be used as nutrients in cells for reproduction of RNA pool [7, 49].

### 3.8. As a Carrier Protein

 The crystallographic studies of ZAG have shown that its groove can bind small hydrophobic ligands [7, 12]. It appears that ZAG via binding to lipid-like molecules in its groove can regulate lipid metabolism. The common determinant with nephritogenic urinary glycoprotein and serum is ZAG; hence, it is concluded that the plasma ZAG may function as a carrier protein of the nephritogenic renal glycoprotein [7].

### 3.9. Tumor Proliferation

 The role of ZAG in malignancies is different. In one hand, ZAG indirectly plays a role in hindering tumor progression via downregulating cyclin-dependent kinase, which regulates G2-M transition, a rate limiting step in the cell cycle. Moreover, ZAG is actively involved in inhibition of tumor growth and proliferation via its linking with immune system [7]. The capacity of macroglobulins such as ZAG for binding hydrolases makes them to prevent the enzyme-mediated tumor invasion [7, 21]. Furthermore, an excess of macroglobulin/hydrolase complexes can activate apoptosis. On the other hand, the progressing tumors express many receptors to macroglobulins such as ZAG, which are carriers of some cytokines and growth factors essential for proliferation [7].

### 3.10. Growth Control

 In Jiaxian red cattle, polymorphism of four loci at the coding region of the ZAG gene has been shown including in Z1, Z2, Z3, and Z4. In one study, Guo et al. showed that Z4 locus can be one of the markers of growth traits in these animals [13].

## 4. Pathologic Roles of ZAG in Human

 The concentration of ZAG in serum has been shown to increase significantly in carcinomas [7]. Hence, it is considered as a good marker for detecting prostate [5, 7, 50], breast, oral, and epidermal carcinomas [7]. The ZAG expression in prostate cancer epithelial cells is inversely associated with Gleason's grade on pathology [6, 50]. This inverse association between Gleason's grade and rising serum ZAG level is similar to that seen with prostate-specific antigen [50]. It is identified that ZAG is increased in the urine samples of patients with bladder cancer [7, 51]. Irmak et al. showed that ZAG is prominent in tumor cells at the bladder tumor invasion front [51]. It appears that ZAG is a clinically important protein, which is directly involved in different types of tumor proliferation [7]; however the exact mechanism by which ZAG results in this proliferation has not been known. This finding is different from other studies which have shown a hindering role for ZAG in tumor proliferation.

 Some studies have demonstrated that low levels of ZAG expression correlate with metastatic potential and a higher risk of recurrence in prostate cancer, suggesting that ZAG can act as an inhibitor of proliferation [6, 16, 52]. Furthermore, some pathological states associated with an increase in epithelial cell proliferation, such as psoriasis and polycystic kidney disease, are accompanied with decreased ZAG expression [16]. It appears that results are different and inverse in the role of ZAG in cell proliferation.

 Moreover, there is a higher level of ZAG in the well-differentiated tumors than that in the moderately or poorly differentiated tumors [5, 7]. It was expressed predominantly in tumors of apocrine and eccrine differentiations [7]; so, ZAG can be considered as a marker of maturation and differentiation.

 Cancer cachexia is characterized by progressive loss of adipose tissue due to increased lipolysis, resulting in the depletion of 85% of the adipose tissue mass and 30% of weight. A potential mediator of this increased lipolysis is a lipid-mobilising factor (LMF) which has been shown to be identical with the plasma ZAG [20]. In cachectic cancer patients, ZAG mRNA is upregulated while leptin mRNA is decreased [1].

 The role of ZAG in cancer cachexia was described by Bing et al. [22]. ZAG can be purified from the urine of patients with cancer cachexia [17]. In cancer cachexia, morphological examination shows marked remodeling of adipose tissue. This remodeling is characterized by shrunken adipocytes with a major reduction in cell size and increased fibrosis in the tissue matrix [53]. It appears that increased lipolysis is a key factor underlying fat loss in cachexia, while inhibition of adipocyte development and lipid deposition may also contribute [54]. In cachexia, both LMF and ZAG can induce lipolysis directly by a cyclic AMP-mediated process, and this is initiated through binding to a *β*3-adrenoreceptor [20]. Fasting levels of serum cortisol have been revealed to be significantly higher in weight-losing patients with stage IV breast cancer than those who have no weight loss [20, 33]. This suggests that the changes in ZAG expression in adipose tissue in cancer cases can be secondary to increase in serum cortisol level [20]. Furthermore, as another mechanism for lipolysis in cachexia, Sanders and Tisdale demonstrated that ZAG can induce uncoupling protein-1 (UCP-1) expression in brown, but not white, adipose tissue, which can be attenuated by *β*3-adrenoreceptor antagonist. Their study showed that ZAG can directly influence UCP expression, which is responsible for lipid utilization during cancer cachexia [26].

 The ZAG is involved in the pathogenesis of proliferative diabetic retinopathy [7, 54]. It is also a biomarker for glomerular disease. It has been revealed that ZAG is among the proteins excreted in urine of microalbuminuria-positive diabetes patients [7]. In cases of chronic hemodialysis, median ZAG serum level is elevated [19]. Jia et al. showed that ZAG can be of potential biomarkers for early noninvasive diagnosis of the acute rejection after renal transplantation [55]. Schmitt et al. showed that aged mouse kidneys displayed a reduced epithelial proliferative reserve due to increased proximal tubular expression of ZAG. Their findings revealed ZAG implication in epithelial proliferative inhibition, which is different from other previous studies that have shown the proliferative role for ZAG. It appears that ZAG can influence the turnover of extracellular matrix components; hence, not only ZAG is a novel regulator of cell proliferation in the aging kidneys and as a modifier of the aging phenotype but also it may be implicated in the regulation of renal interstitial homeostasis [16].

 The ZAG is a marker for response to thalidomide-based therapy in patients with multiple myeloma. Rajpal et al. showed that ZAG along with vitamin D-binding protein, serum amyloid-A protein, and *β*2-microglobulin has significantly higher concentration in nonresponders to thalidomide-based induction therapy compared to responders, while haptoglobin has a lower concentration [56].

 The ZAG is one of the biomarkers in multiple sclerosis. Tumani et al. have shown that the level of ZAG in cerebrospinal fluid is downregulated in the episode of multiple sclerosis [57].

 Findings have showed that ZAG can be as a biomarker for human metabolic alterations [58]. The ZAG is decreased in liver fibrosis in hepatitis C and cirrhosis, which suggests the role of ZAG in their pathogenesis [7]. Regarding these findings, it appears that upregulation of ZAG can be a promising therapeutic target for metabolic syndrome [30].

## 5. An Overview of Vitiligo

 Vitiligo is the most common depigmenting skin disorder [59], characterized by acquired, progressive, and circumscribed amelanosis of the skin and hair [60]. It commonly begins in childhood or young adulthood. Its incidence in males and females is equal [61, 62].

 The pathogenesis of this disorder is uncertain, but it appears to be dependent on the interaction of genetic, immunological, and neurological factors [8]. The melanocytes in vitiligo are degenerated and seem to be replaced by Langerhans cells [8, 63]. The linkage signals on chromosomes 1, 7, and 17 in Caucasian families with generalized vitiligo and associated autoimmune diseases have been reported [64, 65]. Some of the suggested pathogeneses include impaired melanocyte migration and/or proliferation, accumulation of toxic compounds [8, 66], infections, and psychological, neural, autoimmune, and autocytotoxic factors [8].

 Many patients with generalized vitiligo have serum autoantibodies and circulating autoreactive T cells against melanocytes and melanocyte components [67]. Moreover, increased levels of soluble interleukin (IL)-2 receptor, IL-6, and IL-8 have been found, which further suggest the role of T-cell activation in the vitiligo pathogenesis [68]. Furthermore, detection of significantly higher expression of IL-6 and tumor necrosis factor (TNF)-*α* in vitiligo skin, compared with normal skin, reveals an imbalance of epidermal cytokines at sites of lesions [8, 69].

 It appears that there may be a relationship between stress and the development of vitiligo, because vitiligo patients tend to have higher scores for anxiety, depression, adjustment disorders, obsessive symptoms, and hypochondria [8, 70].

 A new theory is emphasizing that depigmentation in vitiligo patches results from a chronic detachment of melanocytes that is proposed to be designated as melanocytorrhagy, which is possibly related to increased susceptibility to mechanical and other types of stresses [8, 71, 72].

 It has been demonstrated that repigmentation occurs in 10–20% of patients [8, 73, 74]. Sun protection of the vitiliginous areas with sunblocks is important [8, 75, 76] for preventing photodamage and lessening the chance of the Koebner phenomenon [8]. Camouflage products and self-tanning dyes can help to improve lesions cosmetically [8, 77].

 Studies have shown that topical steroids and narrowband UVB monotherapy are the most effective and safest forms of treatment for localized and generalized vitiligo, respectively [8, 77]. Some of other important treatments of vitiligo include photochemotherapy, intralesional and oral steroids, calcipotriol, topical fluorouracil, topical minoxidil, oral L-phenylalanine, surgical producers, 308 nm excimer laser, and homeopathy [8]. In one clinical trial, we have demonstrated that zinc also is effective in the treatment of vitiligo [61].

## 6. Probable Association between ZAG and Vitiligo

 For the first time, we pointed the probable association which might be present between ZAG and vitiligo [8, 64]. It appears that most of the suggested pathogeneses of vitiligo can be attributed to decrease in ZAG as follows:

- Studies have revealed that ZAG as a keratinocyte-derived factor influences melanocyte proliferation and dendricity [7, 43, 44]. Moreover, ZAG can be considered as a marker of maturation and differentiation of cells [7]. Hence, in vitiligo, the lack of ZAG is expected to inhibit melanocyte proliferation, and if we consider the Langerhans cells (placed instead of melanocytes in vitiligo sites) as poorly differentiated melanocytes, this impairing in the process of maturation or differentiation of melanocytes can be attributed to the lack of ZAG.
- Some patients with vitiligo have serum autoantibodies and circulating autoreactive T cells against melanocytes and melanocyte components [68]. Studies have shown that ZAG is effective in immunoregulation [7, 18, 45, 46]. Therefore, ZAG can be effective in prevention of vitiligo by immunoregulation.
- It has been revealed that ZAG is effective in attaining the properties of cell adhesion between cells and extracellular matrices [7]. We know that a chronic detachment of melanocytes has been suggested as a recently identified pathogenesis for vitiligo [8, 71, 72]. Hence, it is expected the melanocyte adhesions to the other cells in epidermis to be impaired in the lack of ZAG.
- In whitish patches of vitiligo, the expression of TNF-*α* is high [8, 69]. Some studies revealed that TNF-*α* can decrease ZAG expression and secretion [4, 25]; hence, increased expression of this cytokine in the site of vitiligo can result in a decrease of ZAG expression.
- Topical steroids are the most effective and safest forms of treatment for vitiligo, especially for the localized one [8, 77]. Studies have shown that steroids are able to increase ZAG expression [20, 22, 23]. Regarding these findings we can conclude that steroids are effective in treating vitiligo via enhancing ZAG.
- As a hypothesis, Bagherani et al. suggested that zinc might be effective in the treatment of vitiligo [64]. Some studies have shown that zinc can precipitate ZAG [8–10]. We can conclude that zinc, by precipitating circulating ZAG in the site of vitiligo, can be effective in treatment of this disease.
- The linkage signals on chromosome 7 in patients with generalized vitiligo and associated autoimmune diseases have been reported [64, 67]. Surprisingly, ZAG gene is located on the chromosome 7 [7]. This association between these findings appears to not be accidental.
- Some findings have shown that the minimal level of ZAG is required for melanin production [7]. Hence, it is expected that lack of ZAG can result in whitish patches of vitiligo. Although this hypothesis is not strongly justifying vitiligo pathogenesis because, in the process of vitiligo, the melanocytes are impaired but not melanogenesis, we can consider this theory for vitiligo-like diseases such as postinflammatory hypopigmentation.

## 7. Conclusion

 Regarding mentioned subjects, the association between vitiligo and ZAG must be confirmed in one robust study in vitro and in vivo. If we can confirm this association, this discovery will result in a big progression in the field of treatment of this debilitating disease.
